# Correction: Lamy et al. Monitoring Solar Radiation UV Exposure in the Comoros. *Int. J. Environ. Res. Public Health* 2021, *18*, 10475

**DOI:** 10.3390/ijerph182413399

**Published:** 2021-12-20

**Authors:** Kévin Lamy, Marion Ranaivombola, Hassan Bencherif, Thierry Portafaix, Mohamed Abdoulwahab Toihir, Kaisa Lakkala, Antti Arola, Jukka Kujanpää, Mikko R. A. Pitkänen, Jean-Maurice Cadet

**Affiliations:** 1 LACy, Laboratoire de L’Atmosphère et des Cyclones, UMR 8105 CNRS, Université de La Réunion, Météo-France, 97744 Saint-Denis, France; marion.ranaivombola@univ-reunion.fr (M.R.); hassan.bencherif@univ-reunion.fr (H.B.); thierry.portafaix@univ-reunion.fr (T.P.); jeanmaurice.cadet@gmail.com (J.-M.C.); 2 School of Chemistry and Physics, University of KwaZulu-Natal, Durban 4041, South Africa; 3 Agence Nationale de l’Aviation Civile et de la Météorologie, Moroni 84646, Comoros; fahardinetoihr@gmail.com; 4 Space and Earth Observation Centre, Finnish Meteorological Institute, 99600 Sodankylä, Finland; kaisa.lakkala@fmi.fi (K.L.); Jukka.Kujanpaa@fmi.fi (J.K.); Mikko.Pitkanen@fmi.fi (M.R.A.P.); 5 Climate Research Programme, Finnish Meteorological Institute, 70211 Kuopio, Finland; antti.arola@fmi.fi

**Text Correction**

There was an error in the original article [1]. The units used for the different values of the Standard Erythemal Doses and Cumulated Standard Erythemal Doses mentioned in the Abstract, Section 3.3 and Section 4 are wrong. The units should be SED instead of J.m^−2^ and SED.min^−1^ instead of J.m^−2^.min^−1^

A correction has been made to the abstract:

As part of the UV-Indien project, a station for measuring ultraviolet radiation and the cloud fraction was installed in December 2019 in Moroni, the capital of the Comoros, situated on the west coast of the island of Ngazidja. A ground measurement campaign was also carried out on 12 January 2020 during the ascent of Mount Karthala, located in the center of the island of Ngazidja. In addition, satellite estimates (Ozone Monitoring Instrument and TROPOspheric Monitoring Instrument) and model outputs (Copernicus Atmospheric Monitoring Service and Tropospheric Ultraviolet Model) were combined for this same region. On the one hand, these different measurements and estimates make it possible to quantify, evaluate, and monitor the health risk linked to exposure to ultraviolet radiation in this region, and, on the other, they help to understand how cloud cover influences the variability of UV-radiation on the ground. The measurements of the Ozone Monitoring Instrument onboard the EOS-AURA satellite, being the longest timeseries of ultraviolet measurements available in this region, make it possible to quantify the meteorological conditions in Moroni and to show that more than 80% of the ultraviolet indices are classified as high and that 60% of these are classified as extreme. The cloud cover measured in Moroni by an All Sky Camera was used to distinguish between the cases of UV index measurements taken under clear or cloudy sky conditions. The ground-based measurements thus made it possible to describe the variability of the diurnal cycle of the UV index and the influence of cloud cover on this parameter. They also permitted the satellite measurements and the results of the simulations to be validated. In clear sky conditions, a relative difference of between 6 and 11% was obtained between satellite or model estimates and ground measurements. The ultraviolet index measurement campaign on Mount Karthala showed maximum one-minute standard erythemal doses at 0.3 SED and very high daily cumulative erythemal doses at more than 80 SED. These very high levels are also observed throughout the year and all skin phototypes can exceed the daily erythemal dose threshold at more than 20 SED.

A correction has been made to Section 3.3, second paragraph:

Standard erythemal doses (SEDs) and cumulative standard erythemal doses (CSEDs) for the day were also calculated in this study. SED and cumulative SED are shown in Figure 7. To compute SED, the erythemal irradiance is accumulated over a one-minute window. In the first panel (Figure 7a), the diurnal cycle of the SED is shown for the day of 12 January 2020, i.e., during the ascent of Mount Karthala. The SED calculated from UVI-RADIO, located in Moroni, is represented by a blue line, SED calculated from UVI-HIKE is an orange line. On the second panel (Figure 7b), the cumulative SED during the day is shown in blue for Moroni and in orange for the hike. The tolerance thresholds defined by the Fitzpatrick scale [52] are also shown for the two extreme phototypes (I and VI). In the last panel (Figure 7c), the cumulative SED over the day (C-SED) is represented for the whole study period calculated from UVI-RADIO and UVI-TUV-CS. The tolerance thresholds are also plotted on this figure. The SEDs observed in Moroni and on Mount Karthala are of the same order of magnitude with maxima reaching values of about 0.25 SED·min^−1^ at Moroni and about 0.3 SED·min^−1^ on Mount Karthala (Figure 7a). Very high SEDs are observed on Mount Karthala between 12:00 and 14:00 local time. These high values are related to the high UVI values observed previously (Figure 6a) and are thus probably due to the increase of UVI by the clouds. The cumulative SED for the day exceeds the Fitzpatrick threshold as early as 09:00 for phototype I at both Moroni and Mount Karthala (Figure 7b). For phototype VI, the threshold is exceeded from around 11:00 local time. The maximum cumulative dose observed on 12 January 2020 was 60 SED on Mount Karthala and 50 SED in Moroni. It was reached at about 15:00 local time in the first case and at about 16:00 local time in the second case. Observations of the daily cumulative SED evolution during the whole study period (Figure 7c), reveals that the thresholds for both phototypes are exceeded almost all year round. Indeed, the cumulative daily doses measured by the ground radiometer (blue crosses) are mostly between 20 SED and 80 SED. The cumulative daily doses modeled in clear sky are between 80 and 40 SED. For no day of the year 2020 did the cumulative doses measured by the radiometer exceed those modeled by TUV. The TUV clear-sky modeling previously showed an overestimation of UVI of about 11%, which explains the discrepancy observed between the two daily cumulative doses measured by the radiometer and those calculated from the TUV modeling. Nonetheless, the high values observed all year long clearly indicate health risks for the exposed population.

A correction has been made to Section 4, third paragraph:

Analysis of the UVI measurements made during the Karthala hike shows a record number of UVI maxima (about 20), which are attributed to the impact of the high altitude and fractional cloud cover. They highlight the greater health risk to the population living and working at medium and high altitudes. The maximum SEDs calculated during this campaign were about 0.30 SED and 0.24 SED over Karthala and Moroni, respectively. Cumulated over the day, we found that the health risk thresholds were exceeded from 09:00 in the morning for phototype I skin and from 11:00 for phototype VI skin, and, over the whole day, the maximum accumulation was 60 SED on Karthala and 50 SED at Moroni. These SED and cumulative SED values (over 50 SED) are close to those obtained in this region of the globe at similar latitudes by [13]. In the northern hemisphere, in Spain, cumulative SED values of over 60 SED were also reported by [55]. The cumulative SEDs over the day for the entire study period presented here show that the health risk thresholds were exceeded in Moroni throughout the year and for any skin type. The high level of cumulative doses throughout the year reinforces the need to monitor UVI in this region and to communicate preventive messages to the general public about the risk of UVR. Few studies exist today to quantify the mortality rate related to this risk, so it is important to study this risk in more detail to understand and prevent it in the future.

**Error in Figure**

In the original article, there was a mistake in Figure 7 as published. The units in the y axis label of Figure 7b,c are wrong. The y axis label of Figure 7b,c should be “Cumulative SED [100 J.m^−2^] instead of “Cumulative SED [J.m^−2^]”. The units in the subtitle of Figure 7a,b are wrong. The subtitle of Figure 7a should be “a) SED on the 12/01/2020” instead of “a) SED on the 12/01/2020 [J.m^−2^]”. The subtitle of Figure 7b should be “b) Cumulative SED on the 12/01/2020” instead of “a) Cumulative SED on the 12/01/2020 [J.m^−2^]”.

The corrected Figure 7 appears below. 

The authors apologize for any inconvenience caused and state that the scientific conclusions are unaffected. The original article has been updated.


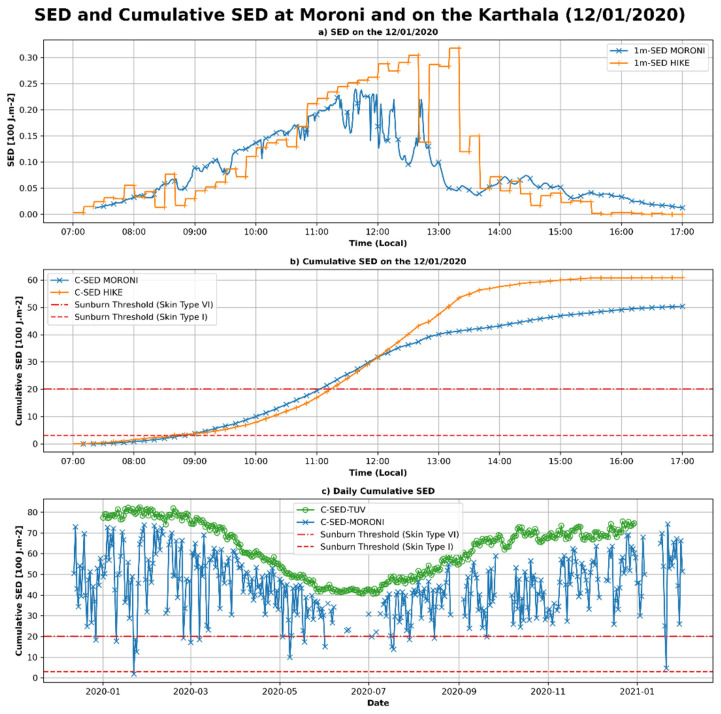


## References

[1] Lamy K., Ranaivombola M., Bencherif H., Portafaix T., Toihir M.A., Lakkala K., Arola A., Kujanpää J., Pitkänen M.R.A., Cadet J.-M. (2021). Monitoring Solar Radiation UV Exposure in the Comoros. Int. J. Environ. Res. Public Health.

